# Reply to Comments on the Study “The Sensitivity, Specificity and Accuracy of Warning Signs in Predicting Severe Dengue, the Severe Dengue Prevalence and its Associated Factors”

**DOI:** 10.3390/ijerph16081380

**Published:** 2019-04-17

**Authors:** Mohd Hanief Ahmad, Mohd Ismail Ibrahim, Zeehaida Mohamed, Nabilah Ismail, Muhammad Amiruddin Abdullah, Rafidah Hanim Shueb, Mohd Nazri Shafei

**Affiliations:** 1 Department of Community Medicine, School of Medical Sciences, Universiti Sains Malaysia, Kubang Kerian, Kota Bharu 16150, Kelantan, Malaysia; haniefahmad@gmail.com (M.H.A.); drnazri@usm.my (M.N.S.); 2 Department of Microbiology & Parasitology, School of Medical Sciences, Hospital Universiti Sains Malaysia, Kubang Kerian, Kota Bharu 16150, Kelantan, Malaysia; zeehaida@usm.my (Z.M.); drnabilah@usm.my (N.I.); amiruddin@usm.my (M.A.A.); hanimkk@usm.my (R.H.S.)

Thank you for the comments received on the article “The Sensitivity, Specificity and Accuracy of Warning Signs in Predicting Severe Dengue, the Severe Dengue Prevalence and its Associated Factors”. These are our responses:

## 1. An Enquiry about Lab-Confirmed Dengue Cases

In our study, the dengue cases were confirmed with an NS1 test kit (Panbio Dengue Early Rapid; Cat. No. 01PF20; manufactured by Standard Diagnostics, Inc, Gyeonggi-do, Republic of Korea) and/or Dengue Serology (IgM/IgG) test kit (Panbio Dengue Duo Cassette; Cat. No. 01PF10; manufactured by Standard Diagnostics, Inc., Gyeonggi-do, Republic of Korea). The work pathway for the dengue confirmatory diagnostic test began by testing the taken blood with an NS1 test kit. If it was positive, no further Dengue Duo Cassette test was required. However, if the NS1 test was negative, a second test with a Dengue Duo Cassette test would be performed. If this second test was positive, then it was considered positive. If the second test negative, the sample would be considered as a confirmed negative sample.

We appreciate the fact that RT-PCR has been the best diagnostic test for dengue in terms of specificity and sensitivity. Moreover, we have discussed this in our discussion (4.1: Prevalence of Severe Dengue), noting the prevalence of severe dengue in other studies using RT-PCR was higher [1]. It would be interesting to know the reason why the proportion of severe dengue would be higher using RT-PCR. Logically, if more dengue cases were detected, the proportion of severe dengue would be more diffuse and lower.

## 2. An Enquiry about Sample Size Calculation

The sample size calculation was done in accordance with the following objectives.

Objective 1: To determine the prevalence of severe dengue (SD) presented to Hospital USM in 2014.

The sample size was calculated using the single proportion formula based on the study done by Thein et al. [1]:

n = (z/Δ)^2^ × (p (1 − p))

= (1.96/0.05)^2^ × 0.165 (1 − 0.165)

= 1536.64 × 0.1377

= 212

Add on 10% for missing/incomplete data = 233, *p based on prevalence of Severe Dengue 16.5% [1]

Objective 2: To determine the predictive values of the warning sign (WS) to diagnose SD among patients admitted into Hospital USM in 2014.

The sample size was calculated using the formula for sensitivity and specificity:

n = (Z_α/2_^2^ × S_N_ (1 − S_N_)/(Δ^2^ P)

(S_N_ = sensitivity, Δ = Absolute precision, P = Prevalence)

n = (Z_α/2_^2^ × S_p_ (1 − S_p_)/(Δ^2^ × (1 − P))

(S_p_ = specificity, Δ = Absolute precision, P = Prevalence), With P (prevalence of severe dengue) = 0.16 [1]

S_N_ = 0.96

S_P_ = 0.55

n for sensitivity was = 406 (including 10% for missing/incomplete data)

n for specificity was = 498 (including 10% for missing/incomplete data)

Objective 3: To determine the factors associated with SD among patients admitted to Hospital USM in 2014.

The sample size was calculated by using PS software to compare two independent proportions (please refer Table 1). The minimum number of samples required to fulfil the objectives of this study was 649 (based on objective 3). Thus, for the purpose of this study, 700 secondary data that fulfil the criteria were chosen and used.

## 3. On Other Comments

We are grateful to our peers who commented on this study. We acknowledge the importance of other variables, including the history of previous infection variable, which we could not capture in our study. This was one of the limitations of our study.

## Figures and Tables

**Table 1:** Sample size calculation using two proportions formula.

Associated Factors	P0*	P1	m	α	Power	n + 10%	Reference*
**Sex (Female)**	0.20	0.40	1	0.01	80%	266	[2]
**High Education**	0.32	0.50	1	0.01	80%	380	[3]
**Diabetes**	0.03	0.25	1	0.01	80%	125	[3]
**Vomiting**	0.46	0.60	1	0.01	80%	649	[2]

P0* Proportion with attribute of interest in control group.
